# Draft Genome Sequence of *Pseudomonas graminis* PDD-13b-3, a Model Strain Isolated from Cloud Water

**DOI:** 10.1128/genomeA.00464-17

**Published:** 2017-06-29

**Authors:** L. Besaury, P. Amato, N. Wirgot, M. Sancelme, A. M. Delort

**Affiliations:** CNRS, Université Clermont Auvergne, Institut de Chimie de Clermont-Ferrand, Clermont-Ferrand, France

## Abstract

The whole genome of *Pseudomonas graminis* PDD-13b-3, a strain of bacteria isolated from cloud water, was sequenced. This showed that this microorganism is equipped with genes that could potentially be involved in its survival in the atmosphere and clouds: those for oxidative stress and carbon starvation responses, DNA repair, and iron uptake.

## GENOME ANNOUNCEMENT

Clouds play a major role in the transformation of atmospheric compounds. They also host living microorganisms, despite high oxidative capacity and UV radiation levels, low temperatures, and rapid shifts of abiotic conditions causing physiological shocks (1–4). Among those microorganisms recovered alive from cloud water, bacteria affiliated with the genus *Pseudomonas* are the most frequently recovered (5). *Pseudomonas graminis* PDD-13b-3 (1) was recovered from a culture of cloud water sampled in July 2004 from Puy de Dôme Mountain’s meteorological station (France), at 1,465 m altitude, on R2A agar medium incubated at 17°C under aerobic conditions. Once purified from the original colony, the strain was identified based on its 16S rRNA gene sequence (GenBank accession number DQ512786). *Pseudomonas graminis* PDD-13b-3 has the ability to develop at low temperature, degrade some organic compounds present in clouds (6), and produce siderophores to satisfy their iron requirements (7); it thus potentially interferes with cloud chemical reactivity. This strain was found to be ice-nucleation negative (8), but it produces biosurfactants known for facilitating spread on surfaces, and perhaps also participating in cell aerosolization and cloud formation (9). *Pseudomonas graminis* PDD-13b-3 appears thus to be a model of cloud water microorganisms. To further understand the specificities of *Pseudomonas graminis* PDD-13b-3, its ability to survive in cloud water, and the implications of its presence in clouds, its whole genome was sequenced and is reported here. Whole-genome shotgun sequencing (2 × 150 bp) was prepared using the Nextera DNA sample preparation kit (Illumina, San Diego, CA, USA), according to the manufacturer’s user guide, and sequenced on an Illumina MiSeq sequencer (MR DNA; Molecular Research, Shallowater, TX, USA). Sequence data files were filtered for quality using FastQC (https://www.bioinformatics.babraham.ac.uk/projects/fastqc/), trimmed using Prinseq-Lite (10), and then *de novo* assembled with SPAdes (11). A total of 102 contigs were generated, with an average coverage of 22.6-fold. The average contig size was 58,931 bp, and the *N*_50_ contig size was 152,216 bp. The size of the assembled genome is 5,686,785 bp, with a G+C content of 60.11%, which are within the ranges of known values for *Pseudomonas* genomes (12).

The draft genome of *Pseudomonas graminis* PDD-13b-3 was annotated using the RAST annotation server (http://rast.nmpdr.org). It contains 61 RNAs and 5,177 protein-coding genes, of which 52% were assigned to a total of 527 SEED subsystems. Among these SEED subsystems, 98 were affiliated with oxidative stress response (encoding NADPH:quinone oxidoreductase, glutathione synthetase, peroxidase, etc.) and 85 with DNA repair processes. Eleven protein-coding genes were related to iron acquisition and metabolism, encoding iron siderophore (pyoverdine) and related sensor and receptor systems. Interestingly, 8 protein-coding genes were associated with carbon starvation. All these could have contributed to *Pseudomonas graminis* PDD-13b-3 survival in clouds.

### Accession number(s).

This whole-genome shotgun project has been deposited in the GenBank database under the accession number MTSB00000000.
